# Oral Health Implications of Obstructive Sleep Apnea: A Literature Review

**DOI:** 10.3390/biomedicines12071382

**Published:** 2024-06-21

**Authors:** Antonino Maniaci, Salvatore Lavalle, Riccardo Anzalone, Antonino Lo Giudice, Salvatore Cocuzza, Federica Maria Parisi, Filippo Torrisi, Giannicola Iannella, Federico Sireci, Gianluca Fadda, Mario Lentini, Edoardo Masiello, Luigi La Via

**Affiliations:** 1 Department of Medicine and Surgery, University of Enna Kore, 94100 Enna, Italy; salvatore.lavalle@unikore.it (S.L.); filippo.torrisi@unikore.it (F.T.); 2 Otorhinolaryngology Section, Biomedicine, Neuroscience and Advanced Diagnostic Department, University of Palermo, 90127 Palermo, Italy; riccardoanzalone1@gmail.com (R.A.); federicosireci@hotmail.it (F.S.); 3 Department of Biomedical and Surgical and Biomedical Sciences, Catania University, 95123 Catania, Italy; antonino.logiudice@unict.it; 4 Department of Medical, Surgical Sciences and Advanced Technologies “GF Ingrassia” ENT Section, University of Catania, 95124 Catania, Italy; s.cocuzza@unict.it (S.C.); federicamariaparisi@gmail.com (F.M.P.); 5 Otorhinolaryngology Department, Sapienza University of Rome, 00161 Rome, Italy; giannicola.iannella@uniroma1.it; 6 Department of Otolaryngology, San Luigi Gonzaga Hospital, University of Turin, 10043 Orbassano, Italy; dott.fadda@gmail.com; 7 ASP Ragusa-Hospital Giovanni Paolo II, 97100 Ragusa, Italy; marlentini@tiscali.it; 8 Department of Radiology, IRCCS San Raffaele Scientific Institute, 20132 Milan, Italy; masiello.edoardo@hsr.it; 9 Department of Anesthesia and Intensive Care, Azienda Ospedaliero Universitaria Policlinico “G.Rodolico-San Marco”, 95123 Catania, Italy; luigilavia7@gmail.com

**Keywords:** obstructive sleep apnea, oral health, bruxism, periodontal disease, temporomandibular joint disorders, oral appliance therapy

## Abstract

Background: Obstructive sleep apnea (OSA) is a prevalent sleep disorder characterized by repeated episodes of partial or complete obstruction of the upper airway during sleep. While the systemic implications of OSA are well documented, the dental consequences are less frequently discussed yet equally significant. This review aims to elucidate the oral health impacts of OSA, emphasizing the importance of interdisciplinary care. Methods: A comprehensive literature search was conducted across several databases to identify studies examining the relationship between OSA and various oral health parameters. The review included observational studies, clinical trials, and systematic reviews published in English up to January 2024. Results: OSA was significantly associated with heightened risks of bruxism, dry mouth, periodontal disease, temporomandibular joint disorders, palatal and dental changes, and alterations in taste sensation. Mouth breathing associated with OSA was a critical factor in exacerbating xerostomia and dental caries. Furthermore, the systemic inflammation induced by OSA appeared to correlate with the severity of periodontal disease. Patients using oral appliance therapy for OSA also showed notable changes in dental occlusion and required ongoing dental monitoring. Conclusions: The findings underscore the bidirectional relationship between OSA and oral health, highlighting the need for dental professionals to be integral participants in the management of OSA. Early dental evaluation and intervention can contribute to the overall health and quality of life of individuals with OSA. The review advocates for the development of clinical guidelines to facilitate the early identification and management of OSA-related oral health issues within dental practice and encourages a collaborative approach to patient care.

## 1. Introduction

**Figure 1:**
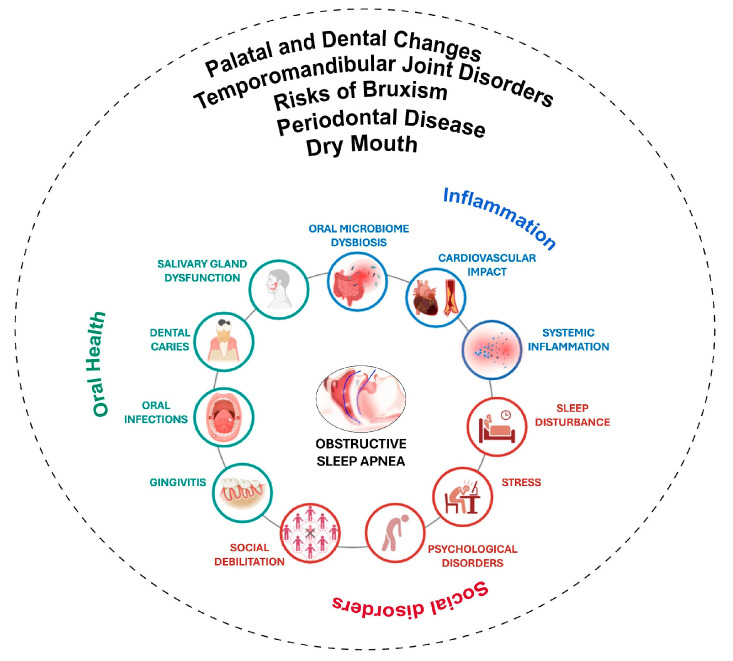
Main alteration in OSA patients and oral diseases. This figure was created with BioRender.com.

Systemic health and oral health are inextricably linked, with oral health disorders frequently reflecting or exacerbating more general health problems [1,2]. The connection between oral health and obstructive sleep apnea (OSA) [3], a sleep-breathing condition marked by repeated partial or total collapse of the upper airway during sleep [4,5,6], is especially clear in human patients. The systemic character of oral health is highlighted by the interactions between OSA and many oral health issues, such as bruxism, xerostomia, periodontal disease, temporomandibular joint disorders, palatal and dental structural changes, and changes in taste perception [7,8,9,10,11,12,13,14]. Particularly noteworthy are the effects of palatal and dental abnormalities on the severity of OSA [15,16]. The nasal cavity may become narrowed by a high-arched, small palate, which can increase the risk of airway resistance and collapse. The typical anatomic relationships inside the oral cavity may also be altered by malocclusion and changes in the location or tone of the soft palate, which can restrict the airway and exacerbate OSA symptoms [17,18]. Furthermore, increased airway blockage during sleep may result from changes in tongue position brought on by changes in tooth structure [19,20]. Furthermore, mouth breathing can worsen dry mouth and increase the risk of dental caries. It is commonly used as a compensatory mechanism in people with OSA due to nasal obstruction [3,21]. This is made worse by the fact that mouth breathing reduces saliva, which guards against tooth decay. The clinical picture is further complicated by the fact that bruxism is also commonly observed in individuals with OSA, which can result in temporomandibular joint problems and pain [7,8,9] (Figure 1). The amount of research in human studies demonstrating these links has increased over the previous 20 years, but there are still few evaluations of the literature that concentrate on the complex interaction between OSA and oral health. By combining the research on the relationships between OSA and various oral health concerns and concentrating on how oral health can vary in relation to OSA, affect the severity of the condition, and affect patient approach, this thorough review sought to close this knowledge gap. Our goal was to highlight the importance of multidisciplinary methods and integrated care that take into account sleep medicine and dental health in order to improve patient outcomes and quality of life. This review focused on the impact of obstructive sleep apnea (OSA) on oral health in human patients.

## 2. Materials and Methods

Our goal in performing this comprehensive study was to compile the body of knowledge regarding the relationship between periodontitis and obstructive sleep apnea (OSA) in human subjects that has been developed over the last 20 years, obtaining the latest and most pertinent information on the subject by narrowing the scope of our search to observational studies, clinical trials, and systematic reviews published. We used keywords associated with obstructive sleep apnea (OSA) and different elements of dental health with Medical Subject Headings (MeSH) terms to perform an extensive literature search across the PubMed, EMBASE, Cochrane Library, and Scopus databases. A number of pertinent terms were combined with the main MeSH term, “Sleep Apnea, Obstructive”, including “Oral Health”, “Bruxism”, “Xerostomia”, “Periodontal Diseases”, “Temporomandibular Joint Disorders”, “Dental Occlusion”, “Palate”, “Taste Disorders”, “Mouth Breathing”, “Dental Carie”, and “Salivation”. These words were purposefully chosen to cover a broad spectrum of possible connections between oral health issues and OSA. Boolean operators such as “AND” and “OR” were employed to hone the search results and make sure the most relevant articles were found. The study features (authors, year, and design), significant findings about the relationship between OSA and other oral health indices, and the hypothesized processes underlying these relationships were among the data retrieved from the included papers. The primary oral health issues linked to OSA, such as dry mouth, periodontal disease, bruxism, temporomandibular joint disorders, palatal and dental changes, and taste disorders, were presented along with supporting data from the reviewed studies in a thematic organization of the data analysis. Furthermore, a thorough analysis was conducted of the interdisciplinary aspects of sleep apnea and dental health, as well as all the participating health specialists.

## 3. Results

The review uncovered an intriguing relationship between oral health and OSA. Our data show that there is a complicated, reciprocal interaction between OSA and a range of oral disorders, rather than the two illnesses existing independently. Gaining this insight is essential to transitioning from symptom management to a patient-centered approach. Mouth breathing is a common adaptation used by OSA patients who are experiencing nasal blockage; it is a clear illustration of this interplay [3,21]. Although at first a compensatory strategy, it causes a host of adverse effects, most notably xerostomia (dry mouth) and heightened susceptibility to dental caries [22,23,24,25]. This emphasizes the necessity of treating the apnea’s subsequent effects on the oral environment in addition to the condition itself. The connection between periodontal disease and OSA strengthens this interdependence. The extensive effects of OSA are highlighted by the finding that the persistent, systemic inflammation linked to the sleep condition appears to accelerate the development of periodontal disease [26,27,28]. This relationship highlights the value of a team approach to patient care that includes dentists and sleep medicine specialists. Beyond these particular cases, our analysis found a variety of oral health problems associated with OSA, such as bruxism, disorders of the temporomandibular joint, changes in the palatal and dental regions, and even taste abnormalities [7,8,9,10,18,19]. The substantial effects that any of these illnesses can have on a person’s quality of life highlight the necessity of early detection and treatment.

### 3.1. Dry Mouth

Xerostomia, often known as dry mouth, is a common yet serious ailment that has been strongly associated with OSA [10,11]. It results from a decrease in salivary flow, which is a frequent occurrence in OSA patients as a result of repeated mouth breathing when the nasal airway becomes blocked during sleep episodes [22,23,24,25]. Over the last twenty years, research has repeatedly demonstrated that xerostomia is not just an uncomfortable symptom but also a factor that can make oral health disorders worse [26,27,28]. (Reference Table 1). Saliva is essential for maintaining dental health because it lubricates the mouth cavity, aids digestion, buffers acids to preserve dental enamel, and acts as an antibacterial to ward against infection [29,30,31,32]. A number of dental issues, such as an elevated risk of dental caries, periodontal disease, and oral infections including candidiasis, can result from OSA-related impaired saliva production [33,34]. If left untreated, the chronicity of dry mouth in OSA patients can also result in an ongoing downward spiral in their oral health. A dry mouth can compromise the soft tissues of the mouth, causing discomfort, ulcers, and trouble using dental prostheses. Additionally, studies have shown that xerostomia can negatively influence a person’s quality of life by impairing speech, changing taste perceptions, and causing halitosis, which can be extremely debilitating for social interactions [35]. Healthcare professionals must advise patients on regular oral hygiene practices that can reduce the risks associated with reduced saliva flow, endorse saliva substitutes or stimulants, and encourage patients to maintain adequate hydration given the complex implications of xerostomia in OSA [36,37,38,39].

### 3.2. Periodontal Disease

**Figure 2:**
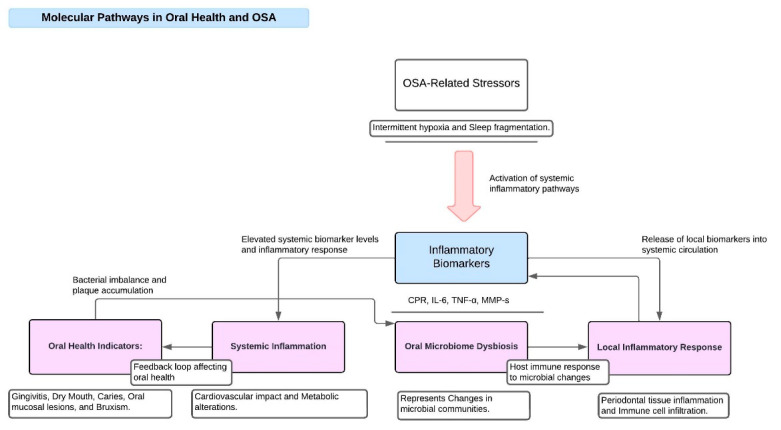
Molecular pathways of inflammation in OSA patients and oral diseases.

Studies conducted over the last 20 years have revealed that, in comparison to the general population, patients with OSA had a higher prevalence of periodontal disease [59]. The scientific literature has increasingly shown a correlation between periodontal disease, a chronic inflammatory disorder that affects the supporting tissues of the teeth, and obstructive sleep apnea (OSA) [40,41,42] (Figure 2). It has been proposed that the increased inflammatory response associated with OSA, which results from sleep fragmentation and intermittent hypoxia, may increase the load of inflammation throughout the body and make people more susceptible to periodontal disease [43,60]. Particularly noteworthy is the reciprocal link between OSA and periodontal disease, in which inflammatory mediators and cytokines are essential for both disorders [42]. Systemic inflammation brought on by OSA has the potential to worsen periodontal inflammation and hasten the loss of alveolar bone and periodontal tissue. A study by Trombone et al. found a correlation between TNF-α levels and several markers of oral health. To be more precise, they discovered positive connections (*p* < 0.01) between TNF-α levels and attachment loss, probing depth, and mean probing depth [44]. A second study by Latorre et al. found a significant correlation between periodontitis and mild OSA. According to this link, pro-inflammatory molecules are activated, and oxidative stress is induced by hypoxia and the periodic reoxygenation characteristic of OSA [45]. On the other hand, inflammatory cytokines generated during periodontal disease, such as TNF-α and interleukins (IL-1, IL-6), could penetrate the systemic circulation and exacerbate the inflammatory condition linked to OSA. According to a study by Jimenez et al., 66.66% of patients with periodontitis also had a diagnosis of severe OSA. This suggests a tendency for individuals suffering from periodontitis to experience severe apnea, even though there was no statistically significant difference found [46]. The three main indicators of periodontal disease—deeper periodontal pockets, clinical attachment loss, and degeneration of the alveolar bone—are more common in OSA patients [61]. Additionally, it has been demonstrated that periodontal disease and OSA increase the risk of tooth loss, which can have major functional and cosmetic consequences for patients [62]. Given that both periodontal disease and OSA are established risk factors for cardiovascular disease (CVD), their association also has consequences for cardiovascular health [63].

Research indicates that maintaining good dental health can lower the risk of cardiovascular events considerably. According to de Oliveira et al.’s research, preserving good oral hygiene with treatments like brushing, professional teeth cleaning, routine dental checkups, and periodontal therapy may help lower the prevalence of CVD [64]. This emphasizes the value of a multidisciplinary approach to patient care, since treating periodontal disease may benefit organ systems other than the mouth. Collaboration between dental and medical experts is necessary for the management of periodontal disease in individuals with OSA [65]. By administering the proper care and stressing the value of upholding proper oral hygiene habits, dentists are essential in the early diagnosis of periodontal disease [66]. On the other hand, Berggren et al. interviewed dentists and dental hygienists employed by the Swedish Public Dental Service and discovered that different dental experts had different experiences with OSA and that possible oral health problems like OSA were not always identified. The explanation for this lack of awareness was given as a lack of understanding of OSA and proven indices for identifying OSA risk. Increasing knowledge of this topic may facilitate better cooperation between dentists and other healthcare providers [67]. In addition, sleep specialists who treat OSA should be aware of the possible consequences for oral health and think about referring patients to dentists when periodontal disease symptoms are evident.

### 3.3. Risks of Bruxism

In recent years, there has been a growing body of scientific literature reporting on the prevalence of bruxism in people with OSA. Some writers have suggested that as many as one in two OSA patients may have this condition [47]. Arousal reactions, anxiety, stress, and certain lifestyle choices are among the common risk factors that may contribute to a complex pathophysiological relationship between OSA and bruxism [48]. It has been proposed that this co-occurrence serves as a compensatory strategy to prevent the pharyngeal collapse that occurs during sleep apneas [47,48,49]. The grinding motion might encourage the airway’s muscles to contract, which could support the airway’s patency [49]. Moreover, studies have demonstrated that bruxism can cause sleep disturbances, which are another characteristic of OSA [50]. As a protective reflex to continue breathing, the arousal response brought on by bruxism may temporarily restore muscular tone in the upper airway and reopen the airway [68]. On the other hand, this activity may also cause sleep fragmentation, which exacerbates the OSA-related general sleep disturbance. According to the literature, treating one ailment may have an impact on another. For instance, Inana et al. suggest that OSA, which is connected to increased sympathetic activity, may be the cause of this issue. Additionally, there are signs that sleep bruxism is associated with impaired brainstem inhibitory circuit modulation [51]. Because bruxism not only impairs tooth health but may also contribute to the exacerbation of OSA symptoms, its detection and treatment are deemed essential to the management of OSA [69]. Because of the high stresses placed on the periodontium, OSA-related bruxism can cause jaw abnormalities, periodontal disease, and increased tooth sensitivity [52]. Therefore, improved screening procedures in both sleep medicine and dentistry are necessary to address the intricate interactions between these two diseases, in addition to multidisciplinary treatment approaches.

### 3.4. Temporomandibular Joint Disorders

Temporomandibular joint disorders (TMD) include a range of disorders affecting the masticatory muscles, jaw joint, and related structures [70]. Regarding the field of oral health problems associated with OSA, TMD has become a highly concerning condition. In human studies, multifaceted factors have been demonstrated to influence the pathophysiological link between TMD and OSA [71]. Repetitive reflexive jaw clenching during apneic episodes is a common symptom of TMD [53]. It is thought to be an unconscious attempt to prevent airway collapse in people with OSA [72]. This can result in pain, muscle exhaustion, and spasm. Further predisposing people to TMD is the negative intrathoracic pressure produced during obstructive episodes, which can result in greater biomechanical stress on the temporomandibular joint [73]. Individuals who suffer from both OSA and concomitant TMD may also experience a wide range of symptoms, such as headaches, jaw pain, and joint noises such as popping or clicking [74]. According to a study by Klasser et al., bruxism is frequently linked to OSA, which can aggravate these symptoms and result in a complex clinical presentation that lowers patients’ quality of life [54]. Furthermore, because oral appliances may worsen TMD symptoms or be less well tolerated by individuals who already have TMD, TMD can affect how well oral appliances work to treat OSA [75]. The results of treating OSA with mandibular advancement device (MAD) treatment have a variety of consequences on TMD outcomes, as per Langaliya’s comprehensive review. Some studies showed a notable decrease in TMD symptoms’ severity and frequency post-MAD treatment, especially with monobloc, hard, and heat-cured appliances. Other research, nevertheless, found no appreciable changes in TMD symptoms [76]. Owing to the increased frequency of TMD in patients with OSA, these individuals must receive comprehensive assessment and management. Dental practitioners should be on the lookout for TMD symptoms in patients who have been diagnosed with OSA or who are suspected of having it. To properly diagnose and treat temporomandibular disorder (TMD), a comprehensive history and clinical examination with a focus on the masticatory muscles and temporomandibular joint are essential. Conservative approaches, including teaching patients self-care techniques, using occlusal splints, jaw exercises, and pain management, are commonly used in the management of TMD in OSA patients [77]. When using oral appliance therapy for OSA, it is important to monitor and modify the device carefully to reduce the chance of exacerbating TMD [78].

### 3.5. Palatal and Dental Changes

In individuals with OSA, the interaction between palatal and dental alterations is important for oral health because it affects both the severity of the condition and the prognosis of the patient [55]. A high-arched and narrow hard palate, which narrows the nasal airway and increases airway resistance and collapsibility, is a common palatal alteration seen in OSA patients [79]. Furthermore, dental issues can worsen the symptoms of OSA. Narrow teeth can cause crowding and alter the occlusal plane, which can alter the tongue’s resting position, cause anomalies in dental occlusion, and ultimately compromise the patency of the airway during sleep [80]. Additionally, research suggests that OSA may result in modifications to dental occlusion. In research by Ishida et al., significant dentoskeletal alterations were identified in individuals with OSA treated with MAD [81]. Tooth position can be influenced by the persistent negative pressure created when breathing against an obstruction, especially in children and teenagers [82]. Malocclusion, which can be brought on by these changes in tooth position, may contribute to the onset or worsening of OSA [83]. Furthermore, continuous positive airway pressure (CPAP) therapy should be considered when managing OSA over the long term [84]. Dental adverse effects from CPAP have been linked to modifications in occlusal relationships and bites. Cephalometric measures in research by Tsuda et al. showed significant alterations, such as reduced maxillary–mandibular discrepancy, anterior maxillary retrusion, setback of the supramentale and chin locations, retroclination of maxillary incisors, and decreased convexity [85]. The pressure that the CPAP mask puts on the maxilla can cause these dental alterations, which may eventually cause teeth to move [86]. Healthcare professionals must conduct a thorough oral examination as part of the OSA assessment because palatal and dental changes have an impact on the condition. When a patient presents with a particular dental or palatal configuration, orthodontists and dentists should be aware of the possibility of OSA and should consider referring the patient to a sleep expert when necessary. Furthermore, it is important to properly plan oral appliance therapy—which is frequently used to treat OSA—to minimize negative dental alterations and maximize airway patency [87]. In some circumstances, orthodontic procedures may help address the anatomical abnormalities that underlie OSA [88].

### 3.6. Taste Disorders

**Figure 3:**
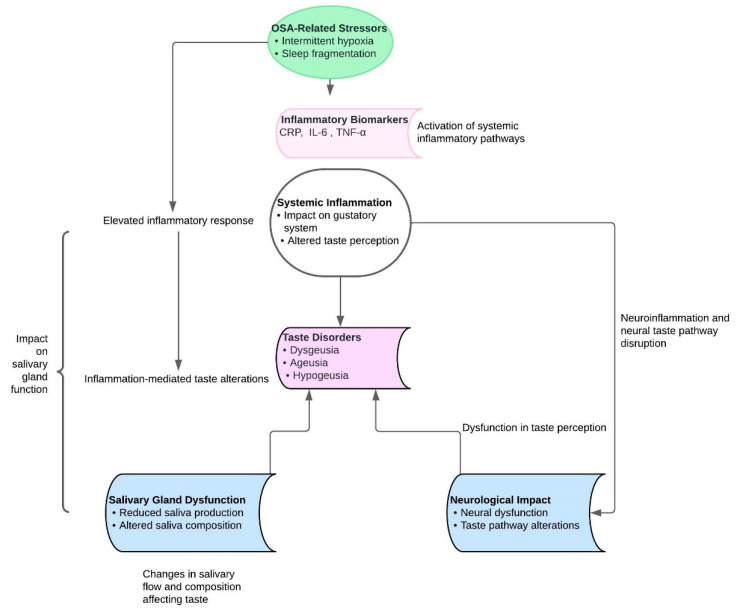
Taste disorders molecular mechanisms in OSA patients.

Changes in taste perception are a less well-known but significant oral health concern in people with OSA [56], particularly considering the potential effects of the chronic intermittent hypoxia that is a feature of OSA on the gustatory system [89]. Yenigun et al.’s case-control investigation revealed a significant difference (*p* = 0.016) in the groups’ smell thresholds during the identification test. Similar substantial differences (*p* < 0.05) were found for the sweet, sour, salty, and bitter taste thresholds between the groups in the tasting test [57]. According to a systematic review by Binary et al., individuals suffering from obstructive sleep apnea (OSA) demonstrate diminished olfactory ability. Moreover, the review indicates that taste abnormalities may potentially be influenced by impairments in scent perception [90]. According to these findings, people with OSA may undergo recurrent oxygen desaturation followed by reoxygenation, which may cause brain alterations that could affect how they perceive flavor (Figure 3) [91]. Taste buds, which are mostly found on the tongue, oversee the detection of flavors such as sweet, sour, bitter, salty, and umami. They are also important for controlling appetite and choosing foods. Dysgeusia, or reduced sensitivity or changed taste perception, is a condition associated with OSA where the hypoxic environment may impact taste bud cell turnover and function [92]. Significant results from an experiment by Liu et al. showed that patients with diverse degrees of OSA severity varied not only in odor thresholds, odor discrimination, and odor identification but also in overall taste score (*p* = 0.004) [58]. This may have an indirect effect on dietary preferences, leading people to select more highly flavored or sweet meals. This could have a negative impact on oral health outcomes, such as periodontal disease and dental caries [57,90,91,92]. Furthermore, by affecting salivary flow, taste changes may have an indirect impact on oral health [93]. Normal taste stimulation causes saliva to flow more readily, which is important for maintaining good dental hygiene since saliva helps wash away food particles and neutralize toxic acids. Reduced salivary production due to a damaged taste receptor can exacerbate dry mouth symptoms and raise the risk of oral illness. The effects of modified taste perception extend beyond one’s bodily well-being [94]. Aside from affecting one’s psychological health and quality of life, taste problems can also result in changes in appetite, a loss of interest in food, and even nutritional inadequacies [95]. The management of taste abnormalities associated with OSA is still developing. The current methods concentrate on treating the underlying OSA, which may entail using oral appliances or CPAP machines to keep the airways open during sleep. According to a recent study by Boerner et al., patients with moderate to severe OSA experienced a considerable improvement in their olfactory and taste performance following three months of nasal CPAP therapy [96]. When the underlying sleep disturbance is addressed, there is evidence that good management of OSA can result in improvements in taste perception, suggesting that there may be a reversible component to the disorder [97]. Walliczek-Dworschak et al.’s investigation, however, showed that while consistent CPAP therapy improved olfactory function in OSA patients, with values returning to normal levels following three months of therapy [98], no discernible effect was seen on taste function.

### 3.7. The Role of the Dentist in Addressing Obstructive Sleep Apnea

In the multidisciplinary treatment of obstructive sleep apnea (OSA), dentists are essential. Their proficiency in dental hygiene enables them to make a substantial contribution to the identification, management, and continuing care of patients with OSA [99,100]. Dentists frequently identify OSA symptoms and indicators in patients before other medical experts. Bruxism, a prevalent symptom of OSA, may be suggested by abfractions, severe tooth wear, and fractures [101]. Other oral signs that can raise the risk of OSA include retrognathia, high-arched palates, swollen tonsils and uvula, and scalloped tongues [102,103], and dentists should be aware of these as well. During routine dental checkups, dentists should ask patients about common OSA symptoms such as loud snoring, daytime sleepiness, morning headaches, and dry mouth [104]. Dentists should promptly send patients with suspected OSA to sleep physicians for a thorough examination that includes a polysomnography sleep study [105]. Custom oral appliances for the treatment of OSA can be produced by dentists. These devices aid in repositioning the tongue, advancing the lower jaw, widening the airway, and minimizing or completely stopping snoring and apnea episodes [106,107]. Patients with OSA, who are more likely to experience oral health problems such as xerostomia and periodontal disease, should get customized oral hygiene advice from dentists [108]. The lifetime and efficacy of dental appliances depend on regular cleaning and upkeep. For the purpose of addressing any possible adverse effects and ensuring that oral appliances are fitted and functioning properly, routine dental examinations are essential [109,110]. Dentists, sleep doctors, otolaryngologists, and other pertinent healthcare professionals must work together to effectively manage OSA [111]. In order to guarantee a thorough and well-coordinated treatment plan, dentists must consult with other professionals involved in the patient’s care [112]. Dentists are in a unique position to be extremely important in treating OSA. Dentists can greatly enhance the quality of life of people with OSA by identifying the symptoms and signs, offering suitable treatment options, and working in conjunction with other medical experts [67,113].

### 3.8. The Function of Maxillofacial Surgeons and Orthodontists

An essential part of the multidisciplinary care of obstructive sleep apnea (OSA) is played by orthodontists and maxillofacial surgeons. Their proficiency in surgical procedures, dentofacial orthopedics, and craniofacial development places them in a prime position to tackle the anatomical causes of OSA [114,115]. Orthodontists possess the necessary skills to recognize and treat skeletal and dental abnormalities that could put a person at risk for OSA. Orthodontists are able to evaluate the influence of craniofacial shape on the upper airway through the use of sophisticated imaging methods and cephalometric analysis [116]. Early orthodontic treatments, such as palatal expansion and functional appliances, can help pediatric patients develop their jaws properly and enhance the size of their airways, which may reduce their chance of developing OSA in the future [117,118]. As a first-line therapy option for mild to moderate OSA, orthodontists are essential to the creation and maintenance of dental appliances for adult OSA patients [106]. Orthodontists create customized mandibular advancement devices (MADs) to move the lower jaw forward, widening the upper airway and decreasing the risk of collapse during sleep [109]. In addition to monitoring for potential adverse effects, including tooth movement or discomfort in the temporomandibular joint, routine follow-up visits with orthodontists are essential to ensuring the best fit, comfort, and effectiveness of this equipment [119]. When conservative methods fail to alleviate severe cases of OSA, maxillofacial surgeons provide surgical solutions to correct underlying anatomical problems. It has been demonstrated that maxillomandibular advancement (MMA) surgery, which involves moving the upper and lower jaws forward, greatly improves airway patency and reduces symptoms of open-mouth breathing (OSA) [120,121]. Maxillofacial surgeons may also employ additional surgical techniques, such as genioglossus advancement and uvulopalatopharyngoplasty (UPPP), to address particular blockage sites [122]. In order to treat OSA comprehensively, cooperation between orthodontists, maxillofacial surgeons, and other medical specialists—such as dentists and sleep medicine specialists—is essential. These experts are able to create individualized treatment programs that cater to the particular requirements and preferences of every patient with OSA by using a coordinated and patient-centered approach [19]. The roles of orthodontists and maxillofacial surgeons in the management of OSA will certainly grow as our understanding of the complex relationship between craniofacial morphology and sleep-disordered breathing deepens, highlighting the significance of their expertise in enhancing patient outcomes and quality of life.

### 3.9. Oral Appliance Therapy and Dental Considerations, and Collaborative Approach

For individuals with OSA, the use of oral appliance therapy—such as mandibular advancement devices, or MADs—is a typical course of treatment [87]. By pushing the jaw forward, these devices increase the patency of the upper airway, which can help lessen OSA symptoms. On the other hand, prolonged usage of these appliances may result in several dental abnormalities that need careful observation by dental specialists. Research has indicated that the utilization of oral appliances may lead to modifications in the temporomandibular joint, tooth movement, and dental occlusion [75,76,78]. For instance, the mandibular forward placement necessary for MADs may cause modifications to the occlusal relationships and bite alignment. This may eventually lead to movement of the teeth, shifts in their positions, and possible problems with the temporomandibular joint (TMJ), including pain, popping, or clicking noises. The quality of life, function, and oral health of the patient may all be significantly impacted by these dental issues. When it comes to the treatment of OSA patients receiving oral appliances, dental specialists are essential. To guarantee the best possible therapeutic results and reduce negative effects on oral health, they should be involved in the selection, fitting, and aftercare of these devices [87]. In human studies, it was demonstrated that it is crucial to keep a close eye on dental alterations, such as shifts in tooth position, bite, and temporomandibular joint function, to spot any problems early and take appropriate action [75,76]. This could entail making modifications to the oral appliance, using extra dental tools (such as occlusal splints), or even making a recommendation to see an orthodontist or a TMJ specialist, among other dental specialists. In order to offer OSA patients complete care and maximize the long-term success of oral appliance therapy, collaboration between sleep medicine specialists and dental professionals is essential. To coordinate treatment, sleep medicine specialists and the patient’s dentist should have regular communication and understanding of the possible dental consequences of oral appliance therapy. On the other hand, dentists should understand how to treat OSA using oral appliances and be able to identify any related dental alterations that might call for therapy. A collaborative care strategy involving dentists, sleep medicine specialists, and other healthcare providers is necessary to address the complex link between OSA and oral health [65,66,67]. The creation of standardized procedures and guidelines for the integrated management of OSA and the oral health issues it causes can help with early detection, prompt referrals, and all-encompassing treatment plans. Promoting this cooperative style of patient care requires interdisciplinary education and collaboration between many healthcare disciplines. Dental practitioners need to be aware of the possible effects OSA may have on dental health and how crucial it is to send patients to sleep medicine specialists when necessary. On the other hand, sleep medicine professionals must be aware of the potential effects on dental health and think about sending patients to dentists for assessment and treatment of associated problems. For people with OSA to receive complete, patient-centered care, a multidisciplinary approach is essential. Together, medical professionals can guarantee that OSA patients’ oral health requirements are met promptly and effectively, improving their general health and quality of life. Fostering this collaborative paradigm of care requires the creation of multidisciplinary education programs and open lines of contact between sleep medicine and dentistry practitioners. A smooth continuum of care for patients with OSA can be ensured by bridging the gap between several healthcare professions through regular case discussions, collaborative patient evaluations, and the development of coordinated treatment regimens. Furthermore, the diagnosis and treatment of oral health problems associated with OSA might be facilitated by the adoption of standardized techniques and recommendations. By giving healthcare professionals a framework to work within, these standardized techniques can guarantee that patients receive consistent, evidence-based care in a variety of clinical settings. The involvement of other healthcare providers, such as primary care physicians, respiratory therapists, and nutritionists, can further enhance the comprehensive management of OSA and its associated oral health consequences, in addition to the collaborative efforts between dental and sleep medicine professionals. This multidisciplinary approach can address the wide range of factors, such as lifestyle, comorbidities, and general health conditions, that contribute to the onset and progression of OSA. By adopting this cooperative approach to patient care, medical professionals can take advantage of their specialized knowledge to offer comprehensive, individualized treatments that are customized to meet the individual needs of every OSA patient. The overall health and quality of life for those impacted by this complicated sleep condition and its oral symptoms can be improved by this interdisciplinary partnership in the long run. It can also lead to improved clinical results, fewer complications, and increased patient satisfaction.

### 3.10. Risky Consequences of Untreated Obstructive Sleep Apnea Syndrome

A multitude of grave health issues have been associated with untreated obstructive sleep apnea (OSA), underscoring the significance of prompt diagnosis and efficient treatment. The adverse effects of OSA on cardiovascular health, cognitive function, and overall quality of life have been repeatedly shown by evidence-based reports [123]. Among the most well-established effects of untreated OSA are cardiovascular problems. OSA substantially raises the risk of hypertension, coronary heart disease, stroke, and cardiovascular mortality, according to a meta-analysis conducted by Wang et al. [124]. In human study, the development of cardiovascular illnesses is aided by endothelial dysfunction, oxidative stress, and systemic inflammation, all of which are brought on by the intermittent hypoxia and elevated sympathetic activity linked to OSA [125]. Untreated OSA has also been connected to neurological diseases and cognitive impairment. According to a systematic review by Leng et al., people with OSA are more likely than people without OSA to experience dementia and cognitive deterioration [126]. Patients with OSA may eventually develop cognitive dysfunction as a result of neuroinflammation, oxidative stress, and structural brain alterations brought on by their persistent sleep disruption and hypoxia [127]. Furthermore, an increased risk of metabolic diseases such as type 2 diabetes and metabolic syndrome has been linked to untreated OSA. Independent of other risk variables, a meta-analysis conducted by Ong et al. revealed that OSA considerably raises the risks of developing type 2 diabetes [128]. The development of metabolic problems can be aided by insulin resistance, glucose intolerance, and the dysregulation of hormones that regulate hunger due to intermittent hypoxia and sleep fragmentation in individuals with OSA [129]. Untreated OSA has an influence on more than just physical health; it frequently causes major deficits in social functioning and quality of life. One common symptom of OSA is excessive daytime sleepiness, which can cause strained interpersonal interactions, lower productivity, and an increased risk of accidents [130]. Furthermore, a higher prevalence of anxiety, depression, and other mental health illnesses has been connected to the chronic sleep deprivation and sleep disruptions caused by OSA [131]. Healthcare providers must place a high priority on the early diagnosis and thorough treatment of OSA due to the variety and gravity of the condition’s consequences if left untreated. Clinicians can help reduce the chance of developing these potentially fatal comorbidities and enhance the general health and well-being of those who are affected by OSA by treating it early and efficiently.

### 3.11. Surgical and Conservative Methods of Treating Obstructive Sleep Apnea

A multidisciplinary approach is used to treat obstructive sleep apnea (OSA), with conservative and surgical options available based on the patient’s unique circumstances and the severity of the condition. Improving airway patency, lowering the frequency of apneic episodes, and easing related symptoms and comorbidities are the main objectives of treating OSA [105]. Oral appliance therapy (OAT), continuous positive airway pressure (CPAP) therapy, and lifestyle adjustments are conservative treatment options for OSA. Patients with mild to moderate OSA may benefit from lifestyle changes such as weight loss, positional treatment, and abstaining from alcohol and sedatives [132]. For moderate to severe OSA, CPAP therapy is the gold-standard treatment because it uses a mask to deliver pressured air to maintain airway patency [133]. However, pain, nasal congestion, and other adverse effects might make it difficult for patients to adhere to CPAP therapy [134]. For patients with mild to severe OSA or those who are intolerant of CPAP, oral appliance therapy (OAT) has become a viable substitute for CPAP [107]. OAT includes repositioning the mandible and tongue using specially made dental devices. The most popular kind of mouth appliance used to treat OSA is a mandibular advancement device (MAD), which has been shown to be effective in lowering the apnea–hypopnea index (AHI) and enhancing sleep quality [135]. Surgical procedures may be explored in cases of severe OSA with notable anatomical anomalies or when conservative approaches are insufficient to control the condition. Depending on the exact location of the obstruction, a surgical technique may be chosen that involves bone or soft tissue changes [136]. One of the most popular surgeries for OSA is uvulopalatopharyngoplasty (UPPP), which involves removing extra soft tissue from the throat [137]. Its long-term effectiveness varies, though, and it can lead to postoperative problems such dysphagia and velopharyngeal insufficiency [120]. The forward displacement of the upper and lower jaws during maxillomandibular advancement (MMA) surgery has been demonstrated to be a highly successful treatment for open bite (OSA), especially in individuals with craniofacial anomalies [138]. AHI is significantly reduced, and sleep quality is improved after MMA surgery because it widens the airway and increases soft tissue tension [121]. But MMA is a significant surgical treatment that calls for specific surgical knowledge and cautious patient selection. To achieve the best results, a mix of conservative and surgical therapies could be required in some circumstances. For instance, in order to keep their OSA under long-term management following surgical procedures, patients might still need to use CPAP or OAT [139]. It is crucial to have routine follow-up appointments with a multidisciplinary team that includes sleep medicine specialists, orthodontists, and surgeons in order to evaluate the effectiveness of treatment, check for lingering symptoms, and modify the treatment plan as needed.

## 4. Conclusions and Future Directions

A compelling multidisciplinary problem arises from the link between obstructive sleep apnea (OSA) and several oral health conditions, including bruxism, dry mouth, periodontal disease, temporomandibular joint abnormalities, palatal and dental changes, and changes in taste perception. The bidirectional nature of these relationships—in which OSA can both cause and worsen oral health problems—has been brought to light by this review. In particular, much of the reported research examined oral health indicators between individuals with OSA and non-OSA controls, offering strong evidence that these problems are more common and severe in OSA patients. Compared with matched controls without sleep-disordered breathing, surveys of OSA patients have consistently reported higher rates of bruxism, xerostomia, periodontal disease, and TMJ abnormalities [10,11,47,48,49,53,54]. Similarly, patients with OSA were found to have more significant changes in palatal morphology, tooth structure, and taste perception than healthy people [55,56,57]. These comparative analyses support the claim that rather than being general societal trends, the oral health problems addressed in this research are specifically related to the presence of OSA. The need for increased awareness and collaboration among dental and sleep medicine specialists in recognizing and addressing oral health comorbidities related to OSA is critical. A stronger focus on collaborative care techniques is required as we move forward. To guarantee that patients receive thorough evaluations and treatment programs that address both OSA and its related oral health manifestations, sleep medicine and dentistry experts must collaborate. To do this, interprofessional education and communication will be essential, as will the creation of standardized protocols that include oral health assessments in the usual examination of OSA patients. To clarify the underlying pathophysiological pathways that connect OSA with oral health issues, more study is required. In order to establish causation and evaluate the long-term effects of OSA treatment on oral health outcomes, longitudinal studies are very crucial. Clinical trials are also required to assess how well different OSA treatment modalities, such as oral appliance therapy and CPAP, work to improve oral health metrics in addition to sleep-related results. In addition, it should be considered that the research that made up this evaluation was carried out across the globe in a variety of geographic regions. This breadth offers insightful information, but it also emphasizes the need to take into account possible differences in risk factors, healthcare access, and health outcomes among other populations. The management and presentation of oral health diseases, including OSA, can be influenced by several factors such as socioeconomic status, cultural customs, and healthcare system architecture. Future studies should clarify these demographic and regional disparities in order to create guidelines and tailored therapies that take into consideration the particular requirements of various patient populations. In addition to addressing population-specific difficulties at the interface of OSA and oral health, collaborative international efforts can aid in identifying common themes and best practices. Another promising area for future research is innovation in therapeutic approaches. More research is necessary to determine whether orthodontic or surgical procedures can alter the palatal and dental structures and, consequently, the severity of OSA. In this way, improvements in the form and functionality of oral appliances could increase their efficacy and lessen adverse consequences such as TMD and tooth alterations. In the end, our analysis emphasizes the necessity of a patient-centered strategy that considers the intricate relationship between dental health and sleep. We may advance toward the more effective care of OSA and related oral health issues by expanding research, clinical practice, and education in this multidisciplinary field. The ultimate goal is to improve patient health and quality of life. Although our findings highlight the value of a multidisciplinary approach to managing open mouth breathing (OSA), it is crucial to recognize this review’s limits. The literature search and study selection procedure for this narrative review was less methodical and thorough than it would have been for a systematic review or meta-analysis. As a result, there is a chance that the results given are more biased and varied. To create a more solid evidence basis, future research should be focused on performing a systematic review using a more thorough search approach and stringent inclusion/exclusion criteria. Furthermore, even though this research has found a number of correlations between OSA and parameters related to oral health, the underlying mechanisms and causal linkages are still largely unknown. To clarify the pathophysiological mechanisms relating oral problems with OSA, future research should make use of biochemical, imaging, and longitudinal methods. Moreover, while the current literature provides valuable insights for animal models and oral health, further research on basic models is needed to confirm these findings in humans subjects.

## Figures and Tables

**Table 1:** Summary of references.

Reference	Author(s)	Year	Key Contents
Xerostomia			
[10,11]	Makeeva IM et al.	2021	Associated with xerostomia, reduced salivary flow, and worsening of oral health disorders
	Apessos I et al.	2020	
[22,23,24,25]	Pico-Orozco J et al.	2020	Decreased salivary flow related to OSA due to mouth breathing during sleep
	Huang Z et al.	2023	
	Huang Z et al.	2023	
	Zhang C et al.	2021	
[26,27,28]	Tanasiewicz M et al.	2016	Demonstration that xerostomia is an uncomfortable symptom that can worsen oral health disorders
	Millsop JW et al.	2017	
	Guggenheimer J et al.	2003	
Periodontal disease			
[33,34]	Téllez-Corral MA et al.	2022	Risks of dental caries, periodontal disease, and oral infections correlated with reduced saliva production in OSA patients
	Téllez-Corral MA et al.	2023	
[39]	Shimazaki Y et al.	2017	Regular oral hygiene practices to reduce risks associated with reduced saliva flow and promote adequate hydration in OSA patients
[40,41,42,43]	Ahmad NE et al.	2013	Correlation between periodontal disease and OSA, with an increased inflammatory response
	Chen Y et al.	2023	
	Keller JJ et al.	2013	
	Nizam N et al.	2015	
Inflammatory cytokines			
[42]	Keller JJ et al.	2013	Essential role of inflammatory cytokines in the pathogenesis of both disorders
[44]	Trombone C et al.	2009	Correlation between TNF-α levels and various indicators of oral health, including worsened attachment loss
[45]	Latorre C et al.	2018	Significant correlation between periodontitis and OSA, with activation of pro-inflammatory molecules
[46]	Arango Jimenez N et al.	2023	Tendency for patients with periodontitis to exhibit more severe obstructive sleep apnea
Bruxism			
[47,48,49]	Li D et al.	2023	Prevalence of bruxism in OSA patients, suggesting a physiological relationship between the two
	Wali SO et al.	2017	
	Alqahtani ND et al.	2018	
[50]	Martynowicz H et al.	2019	Bruxism as a potential cause of sleep disturbances
[51]	Inana R et al.	2017	Association of bruxism with increased sympathetic activity and compromised modulation of the brainstem inhibitory circuit
[52]	Lee JH et al.	2023	Harmful effects of bruxism related to periodontal health and tooth sensitivity
Temporomandibular Joint Disorders Palatal and Dental Changes			
[53]	Ning R et al.	2023	Common symptom of TMD correlated with obstructive sleep apnea
[54]	Klasser GD et al.	2015	Frequent association between bruxism and OSA, with an impact on the patient’s quality of life
[55]	Ciavarella D et al.	2023	Importance of palatal and dental alterations in the severity and course of OSA
Taste Disorders			
[56]	Magliulo G et al.	2018	Changes in taste perception as a significant concern in OSA
[57]	Yenigun A et al.	2019	Significant differences in olfactory and taste perception tests between individuals with and without OSA
[58]	Liu Y et al.	2020	Significant variations in taste perception among patients with different severities of OSA

## Data Availability

All the data reported are present on the PubMed web database.

## References

[1] Sampaio-Maia B. (2016). The Oral Microbiome in Health and Its Implication in Oral and Systemic Diseases. Adv. Appl. Microbiol..

[2] He J., Li Y., Cao Y., Xue J., Zhou X. (2015). The oral microbiome diversity and its relation to human diseases. Folia Microbiol..

[3] Suzuki S., Kojima Y., Takayanagi A., Yoshino K., Ishizuka Y., Satou R., Takahashi N., Tazaki M., Kamijo H., Sugihara N. (2016). Relationship between Obstructive Sleep Apnea and Self-assessed Oral Health Status: An Internet Survey. Bull. Tokyo Dent. Coll..

[4] Lévy P., Kohler M., McNicholas W.T., Barbé F., McEvoy R.D., Somers V.K., Lavie L., Pépin J.L. (2015). Obstructive sleep apnoea syndrome. Nat. Rev. Dis. Primers.

[5] Lv R., Liu X., Zhang Y., Dong N., Wang X., He Y., Yue H., Yin Q. (2023). Pathophysiological mechanisms and therapeutic approaches in obstructive sleep apnea syndrome. Signal Transduct. Target. Ther..

[6] Lee J.J., Sundar K.M. (2021). Evaluation and Management of Adults with Obstructive Sleep Apnea Syndrome. Lung.

[7] Lopes A.J.d.C., Cunha T.C.A., Monteiro M.C.M., Serra-Negra J.M., Cabral L.C., Júnior P.C.S. (2020). Is there an association between sleep bruxism and obstructive sleep apnea syndrome? A systematic review. Sleep Breath..

[8] Ferreira N.M., Dos Santos J.F., dos Santos M.B., Marchini L. (2015). Sleep bruxism associated with obstructive sleep apnea syndrome in children. Cranio.

[9] Alshahrani A.A., Alshadidi A.A.F., Alamri M.A.A., Alamri A.A.A., Alshehri A.H.J., Cicciù M., Isola G., Minervini G. (2023). Prevalence of bruxism in obstructive sleep apnea syndrome (OSAS) patients: A systematic review conducted according to PRISMA guidelines and the Cochrane handbook for systematic reviews of interventions. J. Oral Rehabil..

[10] Makeeva I.M., Budina T.V., Turkina A.Y., Poluektov M.G., Kondratiev S.A., Arakelyan M.G., Signore A., Amaroli A. (2021). Xerostomia and hyposalivation in patients with obstructive sleep apnoea. Clin. Otolaryngol..

[11] Apessos I., Andreadis D., Steiropoulos P., Tortopidis D., Angelis L. (2020). Investigation of the relationship between sleep disorders and xerostomia. Clin. Oral Investig..

[12] Molina A., Huck O., Herrera D., Montero E. (2023). The association between respiratory diseases and periodontitis: A systematic review and meta-analysis. J. Clin. Periodontol..

[13] Lembo D., Caroccia F., Lopes C., Moscagiuri F., Sinjari B., D’Attilio M. (2021). Obstructive Sleep Apnea and Periodontal Disease: A Systematic Review. Medicina.

[14] Manfredini D., Thomas D.C., Lobbezoo F. (2023). Temporomandibular Disorders Within the Context of Sleep Disorders. Dent. Clin. N. Am..

[15] Singhal P., Gupta R., Sharma R., Mishra P. (2014). Association of naso-oro-pharyngeal structures with the sleep architecture in suspected obstructive sleep apnea. Indian J. Otolaryngol. Head Neck Surg..

[16] Zhao T., Ngan P., Hua F., Zheng J., Zhou S., Zhang M., Xiong H., He H. (2018). Impact of pediatric obstructive sleep apnea on the development of Class II hyperdivergent patients receiving orthodontic treatment: A pilot study. Angle Orthod..

[17] Triplett W.W., Lund B.A., Westbrook P.R., Olsen K.D. (1989). Obstructive sleep apnea syndrome in patients with class II malocclusion. Mayo Clin. Proc..

[18] Nabiev F.K., Dobrodeev A.S., Libin P.V., Kotov I.I. (2014). Diagnostics and treatment of patients with II class malocclusion associated with obstructive sleep apnea syndrome. Stomatologiia.

[19] Neelapu B.C., Kharbanda O.P., Sardana H.K., Balachandran R., Sardana V., Kapoor P., Gupta A., Vasamsetti S. (2017). Craniofacial and upper airway morphology in adult obstructive sleep apnea patients: A systematic review and meta-analysis of cephalometric studies. Sleep Med. Rev..

[20] Kang J.H., Kim H.J., Song S.I. (2022). Obstructive sleep apnea and anatomical structures of the nasomaxillary complex in adolescents. PLoS ONE.

[21] Tranfić Duplančić M., Pecotić R., Lušić Kalcina L., Pavlinac Dodig I., Valić M., Roguljić M., Rogić D., Lapić I., Grdiša K., Peroš K. (2022). Salivary parameters and periodontal inflammation in obstructive sleep apnoea patients. Sci. Rep..

[22] Pico-Orozco J., Carrasco-Llatas M., Silvestre F.J., Silvestre-Rangil J. (2020). Xerostomia in patients with sleep apnea-hypopnea syndrome: A prospective case-control study. J. Clin. Exp. Dent..

[23] Huang Z., Zhou N., Chattrattrai T., van Selms M.K.A., de Vries R., Hilgevoord A.A.J., de Vries N., Aarab G., Lobbezoo F. (2023). Associations between snoring and dental sleep conditions: A systematic review. J. Oral Rehabil..

[24] Huang Z., Zhou N., Lobbezoo F., Almeida F.R., Cistulli P.A., Dieltjens M., Huynh N.T., Kato T., Lavigne G.J., Masse J.F. (2023). Dental sleep-related conditions and the role of oral healthcare providers: A scoping review. Sleep Med. Rev..

[25] Zhang C., Shen Y., Liping F., Ma J., Wang G.F. (2021). The role of dry mouth in screening sleep apnea. Postgrad. Med. J..

[26] Tanasiewicz M., Hildebrandt T., Obersztyn I. (2016). Xerostomia of Various Etiologies: A Review of the Literature. Adv. Clin. Exp. Med..

[27] Millsop J.W., Wang E.A., Fazel N. (2017). Etiology, evaluation, and management of xerostomia. Clin. Dermatol..

[28] Guggenheimer J., Moore P.A. (2003). Xerostomia: Etiology, recognition and treatment. J. Am. Dent. Assoc..

[29] Melvin J.E. (1991). Saliva and dental diseases. Curr. Opin. Dent..

[30] Farnaud S.J., Kosti O., Getting S.J., Renshaw D. (2010). Saliva: Physiology and diagnostic potential in health and disease. Sci. World J..

[31] Kaplan M.D., Baum B.J. (1993). The functions of saliva. Dysphagia.

[32] Edgar W.M. (1992). Saliva: Its secretion, composition and functions. Br. Dent. J..

[33] Téllez-Corral M.A., Herrera-Daza E., Cuervo-Jimenez H.K., Arango-Jimenez N., Morales-Vera D.Z., Velosa-Porras J., Latorre-Uriza C., Escobar-Arregoces F.M., Hidalgo-Martinez P., Cortés M.E. (2022). Patients with obstructive sleep apnea can favor the predisposing factors of periodontitis by the presence of *P. melaninogenica* and *C. albicans*, increasing the severity of the periodontal disease. Front. Cell. Infect. Microbiol..

[34] Téllez Corral M.A., Daza E.H., Jimenez N.A., Morales Vera D.Z., Velosa Porras J., Latorre Uriza C., Escobar Arregoces F.M., Martinez P.H., Cortés M.E., Otero L. (2023). Biomarkers for the severity of periodontal disease in patients with obstructive sleep apnea:IL-1 β, IL-6, IL-17A, and IL-33. Heliyon.

[35] Li Z., Li J., Fu R., Liu J., Wen X., Zhang L. (2023). Halitosis: Etiology, prevention, and the role of microbiota. Clin. Oral Investig..

[36] Edgar W.M., Higham S.M., Manning R.H. (1994). Saliva stimulation and caries prevention. Adv. Dent. Res..

[37] Wick J.Y. (2007). Xerostomia: Causes and treatment. Consult. Pharm..

[38] van der Reijden W.A., Veerman E.C., van Nieuw Amerongen A. (1993). Speeksel en speekselsubstituten [Saliva and saliva substitutes]. Ned. Tijdschr. Tandheelkd..

[39] Shimazaki Y., Fu B., Yonemoto K., Akifusa S., Shibata Y., Takeshita T., Ninomiya T., Kiyohara Y., Yamashita Y. (2017). Stimulated salivary flow rate and oral health status. J. Oral Sci..

[40] Ahmad N.E., Sanders A.E., Sheats R., Brame J.L., Essick G.K. (2013). Obstructive sleep apnea in association with periodontitis: A case-control study. J. Dent. Hyg..

[41] Chen Y., Metz J.E., Gao H., Gao X. (2023). Association between obstructive sleep apnea and periodontitis in Chinese male adults: A cross-sectional study. J. Prosthet. Dent..

[42] Keller J.J., Wu C.S., Chen Y.H., Lin H.C. (2013). Association between obstructive sleep apnoea and chronic periodontitis: A population-based study. J. Clin. Periodontol..

[43] Nizam N., Basoglu O.K., Tasbakan M.S., Holthöfer A., Tervahartiala T., Sorsa T., Buduneli N. (2015). Do salivary and serum collagenases have a role in an association between obstructive sleep apnea syndrome and periodontal disease? A preliminary case-control study. Arch. Oral Biol..

[44] Trombone A.P.F., Cardoso C.R., Repeke C.E., Ferreira S.B., Martins W., Campanelli A.P., Avila-Campos M.J., Trevilatto P.C., Silva J.S., Garlet G.P. (2009). Tumor necrosis factor-α-308G/A single nucleotide polymorphism and red-complex periodontopathogens are independently associated with increased levels of tumor necrosis factor-alpha in diseased periodontal tissues. J. Periodontal Res..

[45] Latorre C., Escobar F., Velosa J., Rubiano D., Hidalgo-Martinez P., Otero L. (2018). Association between obstructive sleep apnea and comorbidities with periodontal disease in adults. J. Indian Soc. Periodontol..

[46] Arango Jimenez N., Morales Vera D.Z., Latorre Uriza C., Velosa-Porras J., Téllez Corral M.A., Escobar Arregocés F.M. (2023). Relationship of obstructive sleep apnea with periodontal condition and its local and systemic risk factors. Clin. Oral Investig..

[47] Li D., Kuang B., Lobbezoo F., de Vries N., Hilgevoord A., Aarab G. (2023). Sleep bruxism is highly prevalent in adults with obstructive sleep apnea: A large-scale polysomnographic study. J. Clin. Sleep Med..

[48] Wali S.O., Abalkhail B., Krayem A. (2017). Prevalence and risk factors of obstructive sleep apnea syndrome in a Saudi Arabian population. Ann. Thorac. Med..

[49] Alqahtani N.D., Algowaifly M.I., Almehizia F.A., Alraddadi Z.A., Al-Sehaibany F.S., Almosa N.A., Albarakati S.F., Bahammam A.S. (2018). The characteristics of dental occlusion in patients with moderate to severe obstructive sleep apnea in Saudi Arabia. Saudi Med. J..

[50] Martynowicz H., Gac P., Brzecka A., Poreba R., Wojakowska A., Mazur G., Smardz J., Wieckiewicz M. (2019). The Relationship between Sleep Bruxism and Obstructive Sleep Apnea Based on Polysomnographic Findings. J. Clin. Med..

[51] Inana R., Benbir G., Karadeniz D., Yavlal F., Kiziltanb M.E. (2017). Sleep bruxism is related to decreased inhibitory control of trigeminal motoneurons, but not with reticulobulbar system. Neurol. Sci..

[52] Lee J.H., Han K., Lee S.Y. (2023). Associations between obstructive sleep apnea and dental pain and chewing discomfort in Korean adults: A nationwide cross-sectional study. Sci. Rep..

[53] Ning R., Chen J., Lu Y., Guo J. (2023). Obstructive sleep apnea: A follow-up program in its relation to temporomandibular joint disorder, sleep bruxism and orofacial pain. BMC Oral Health.

[54] Klasser G.D., Rei N., Lavigne G.J. (2015). Sleep bruxism etiology: The evolution of a changing paradigm. J. Can. Dent. Assoc..

[55] Ciavarella D., Campobasso A., Conte E., Burlon G., Guida L., Montaruli G., Cassano M., Laurenziello M., Illuzzi G., Tepedino M. (2023). Correlation between dental arch form and OSA severity in adult patients: An observational study. Prog. Orthod..

[56] Magliulo G., De Vincentiis M., Iannella G., Ciofalo A., Pasquariello B., Manno A., Angeletti D., Polimeni A. (2018). Olfactory evaluation in obstructive sleep apnoea patients. ACTA Otorhinolaryngol. Ital..

[57] Yenigun A., Degirmenci N., Goktas S.S., Dogan R., Ozturan O. (2019). Investigation of smell and taste function in patients with obstructive sleep apnoea syndrome. J. Laryngol. Otol..

[58] Liu Y., Fang F., Zhan X., Yao L., Wei Y. (2020). The impact of obstructive apnea sleep syndrome on chemical function. Sleep Breath..

[59] Bianchi E., Segù M., Toffoli A., Razzini G., Macaluso G.M., Manfredi E. (2024). Relationship between periodontal disease and obstructive sleep apnea in adults: A systematic review. Dent. Res. J..

[60] Loke W., Girvan T., Ingmundson P., Verrett R., Schoolfield J., Mealey B.L. (2015). Investigating the association between obstructive sleep apnea and periodontitis. J. Periodontol..

[61] Kim S.R., Son M., Kim Y.R. (2023). Risk of chronic periodontitis in patients with obstructive sleep apnea in Korea: A nationwide retrospective cohort study. Epidemiol. Health.

[62] Takedachi M., Shimabukuro Y., Sawada K., Koshimizu M., Shinada K., Asai H., Mizoguchi A., Hayashi Y., Tsukamoto A., Miyago M. (2022). Evaluation of periodontitis-related tooth loss according to the new 2018 classification of periodontitis. Sci. Rep..

[63] Herrera D., Sanz M., Shapira L., Brotons C., Chapple I., Frese T., Graziani F., Hobbs F.D.R., Huck O., Hummers E. (2023). Association between periodontal diseases and cardiovascular diseases, diabetes and respiratory diseases: Consensus report of the Joint Workshop by the European Federation of Periodontology (EFP) and the European arm of the World Organization of Family Doctors (WONCA Europe). J. Clin. Periodontol..

[64] de Oliveira C., Watt R., Hamer M. (2010). Toothbrushing, inflammation, and risk of cardiovascular disease: Results from Scottish Health Survey. BMJ.

[65] Herrera D., Sanz M., Shapira L., Brotons C., Chapple I., Frese T., Graziani F., Hobbs F.D.R., Huck O., Hummers E. (2024). Periodontal diseases and cardiovascular diseases, diabetes, and respiratory diseases: Summary of the consensus report by the European Federation of Periodontology and WONCA Europe. Eur. J. Gen. Pract..

[66] Yonel Z., Sharma P. (2017). The Role of the Dental Team in the Prevention of Systemic Disease: The Importance of Considering Oral Health As Part of Overall Health. Prim. Dent. J..

[67] Berggren K., Broström A., Firestone A., Wright B., Josefsson E., Lindmark U. (2022). Oral health problems linked to obstructive sleep apnea are not always recognized within dental care-As described by dental professionals. Clin. Exp. Dent. Res..

[68] Schames S.E., Schames J., Schames M., Chagall-Gungur S.S. (2012). Sleep bruxism, an autonomic self-regulating response by triggering the trigeminal cardiac reflex. J. Calif. Dent. Assoc..

[69] González González A., Montero J., Gómez Polo C. (2023). Sleep Apnea-Hypopnea Syndrome and Sleep Bruxism: A Systematic Review. J. Clin. Med..

[70] Palmer J., Durham J. (2021). Temporomandibular disorders. BJA Educ..

[71] Magalhães B.G., Freitas J.L.d.M., Barbosa A.C.d.S., Gueiros M.C.S.N., Gomes S.G.F., Rosenblatt A., Júnior A.d.F.C. (2018). Temporomandibular disorder: Otologic implications and its relationship to sleep bruxism. Braz. J. Otorhinolaryngol..

[72] Durán-Cantolla J., Alkhraisat M.H., Martínez-Null C., Aguirre J.J., Guinea E.R., Anitua E. (2015). Frequency of obstructive sleep apnea syndrome in dental patients with tooth wear. J. Clin. Sleep Med..

[73] Alessandri-Bonetti A., Bortolotti F., Moreno-Hay I., Michelotti A., Cordaro M., Alessandri-Bonetti G., Okeson J.P. (2019). Effects of mandibular advancement device for obstructive sleep apnea on temporomandibular disorders: A systematic review and meta-analysis. Sleep Med. Rev..

[74] Bartolucci M.L., Bortolotti F., Pelligra I., Stipa C., Sorrenti G., Incerti-Parenti S., Alessandri-Bonetti G. (2023). Prevalence of temporomandibular disorders in adult obstructive sleep apnoea patients: A cross-sectional controlled study. J. Oral Rehabil..

[75] Francis C.E., Quinnell T. (2021). Mandibular Advancement Devices for OSA: An Alternative to CPAP?. Pulm. Ther..

[76] Langaliya A., Alam M.K., Hegde U., Panakaje M.S., Cervino G., Minervini G. (2023). Occurrence of Temporomandibular Disorders among patients undergoing treatment for Obstructive Sleep Apnoea Syndrome (OSAS) using Mandibular Advancement Device (MAD): A Systematic Review conducted according to PRISMA guidelines and the Cochrane handbook for systematic reviews of interventions. J. Oral Rehabil..

[77] Gil-Martínez A., Paris-Alemany A., López-de-Uralde-Villanueva I., La Touche R. (2018). Management of pain in patients with temporomandibular disorder (TMD): Challenges and solutions. J. Pain Res..

[78] Sutherland K., Cistulli P.A. (2019). Oral Appliance Therapy for Obstructive Sleep Apnoea: State of the Art. J. Clin. Med..

[79] Kecik D. (2017). Three-dimensional analyses of palatal morphology and its relation to upper airway area in obstructive sleep apnea. Angle Orthod..

[80] Bajrovic N., Nakas E., Dzemidzic V., Tiro A. (2021). The Link Between Obstructive Sleep Apnea and Orthodontic Anomalies in Obese Adult Population. Mater Sociomed..

[81] Ishida E., Kunimatsu R., Medina C.C., Iwai K., Miura S., Tsuka Y., Tanimoto K. (2022). Dental and Occlusal Changes during Mandibular Advancement Device Therapy in Japanese Patients with Obstructive Sleep Apnea: Four Years Follow-Up. J. Clin. Med..

[82] American Academy of Pediatric Dentistry (2017). Management of the Developing Dentition and Occlusion in Pediatric Dentistry. Pediatr. Dent..

[83] Manrikyan G.E., Vardanyan I.F., Markaryan M.M., Manrikyan M.E., Badeyan E.H., Manukyan A.H., Gevorgyan M.A., Khachatryan S.G. (2023). Association between the Obstructive Sleep Apnea and Cephalometric Parameters in Teenagers. J. Clin. Med..

[84] Lavalle S., Masiello E., Iannella G., Magliulo G., Pace A., Lechien J.R., Calvo-Henriquez C., Cocuzza S., Parisi F.M., Favier V. (2024). Unraveling the Complexities of Oxidative Stress and Inflammation Biomarkers in Obstructive Sleep Apnea Syndrome: A Comprehensive Review. Life.

[85] Tsuda H., Almeida F.R., Tsuda T., Moritsuchi Y., Lowe A.A. (2010). Craniofacial changes after 2 years of nasal continuous positive airway pressure use in patients with obstructive sleep apnea. Chest.

[86] Ghadiri M., Grunstein R.R. (2020). Clinical side effects of continuous positive airway pressure in patients with obstructive sleep apnoea. Respirology.

[87] Johal A., Hamoda M.M., Almeida F.R., Marklund M., Tallamraju H. (2023). The role of oral appliance therapy in obstructive sleep apnoea. Eur. Respir. Rev..

[88] Fagundes N.C.F., Perez-Garcia A., Graf D., Flores-Mir C., Heo G. (2022). Orthodontic interventions as a management option for children with residual obstructive sleep apnea: A cohort study protocol. BMJ Open..

[89] Sforza E., Roche F. (2016). Chronic intermittent hypoxia and obstructive sleep apnea: An experimental and clinical approach. Hypoxia.

[90] Binar M., Gokgoz M.C. (2021). Olfactory function in patients with obstructive sleep apnea and the effect of positive airway pressure treatment: A systematic review and meta-analysis. Sleep Breath..

[91] Sahib A., Roy B., Kang D., Aysola R.S., Wen E., Kumar R. (2022). Relationships between brain tissue damage, oxygen desaturation, and disease severity in obstructive sleep apnea evaluated by diffusion tensor imaging. J. Clin. Sleep Med..

[92] Lin H.C., Hwang M.S., Liao C.C., Friedman M. (2016). Taste disturbance following tongue base resection for OSA. Laryngoscope.

[93] Uchida H., Ovitt C.E. (2022). Novel impacts of saliva with regard to oral health. J. Prosthet. Dent..

[94] Müller F., Chebib N., Maniewicz S., Genton L. (2023). The Impact of Xerostomia on Food Choices—A Review with Clinical Recommendations. J. Clin. Med..

[95] Invitto S., Calcagnì A., Piraino G., Ciccarese V., Balconi M., De Tommaso M., Toraldo D.M. (2019). Obstructive sleep apnea syndrome and olfactory perception: An OERP study. Respir. Physiol. Neurobiol..

[96] Boerner B., Tini G.M., Fachinger P., Graber S.M., Irani S. (2017). Significant improvement of olfactory performance in sleep apnea patients after three months of nasal CPAP therapy—Observational study and randomized trial. PLoS ONE.

[97] Wallace E.S., Carberry J.C., Toson B., Eckert D.J. (2022). A systematic review and meta-analysis of upper airway sensation in obstructive sleep apnea—Implications for pathogenesis, treatment and future research directions. Sleep Med. Rev..

[98] Walliczek-Dworschak U., Cassel W., Mittendorf L., Pellegrino R., Koehler U., Güldner C., Dworschak P.O.G., Hildebrandt O., Daniel H., Günzel T. (2017). Continuous positive air pressure improves orthonasal olfactory function of patients with obstructive sleep apnea. Sleep Med..

[99] Lobbezoo F., Ahlberg J., Manfredini D., Winocur E. (2012). Are bruxism and the bite causally related?. J. Oral Rehabil..

[100] Lobbezoo F., Aarab G. (2019). Increasing the visibility of dental sleep medicine. Sleep Med. Clin..

[101] Koyano K., Tsukiyama Y., Ichiki R., Kuwata T. (2008). Assessment of bruxism in the clinic. J. Oral Rehabil..

[102] Ruangsri S., Jorns T.P., Puasiri S., Luecha T., Chaithap C., Sawanyawisuth K. (2016). Which oropharyngeal factors are significant risk factors for obstructive sleep apnea? An age-matched study and dentist perspectives. Nat. Sci. Sleep.

[103] Al-Jewair T.S., Al-Jasser R., Almas K. (2015). Periodontitis and obstructive sleep apnea’s bidirectional relationship: A systematic review and meta-analysis. Sleep Breath..

[104] Jonas D.E., Amick H.R., Feltner C., Weber R.P., Arvanitis M., Stine A., Lux L., Middleton J.C., Voisin C., Harris R.P. (2017). Screening for Obstructive Sleep Apnea in Adults: An Evidence Review for the U.S. Preventive Services Task Force.

[105] Epstein L.J., Kristo D., Strollo P.J., Friedman N., Malhotra A., Patil S.P., Ramar K., Rogers R., Schwab R.J., Weaver E.M. (2009). Adult Obstructive Sleep Apnea Task Force of the American Academy of Sleep Medicine. Clinical guideline for the evaluation, management and long-term care of obstructive sleep apnea in adults. J. Clin. Sleep Med..

[106] Ramar K., Dort L.C., Katz S.G., Lettieri C.J., Harrod C.G., Thomas S.M., Chervin R.D. (2015). Clinical Practice Guideline for the Treatment of Obstructive Sleep Apnea and Snoring with Oral Appliance Therapy: An Update for 2015. J. Clin. Sleep Med..

[107] Sutherland K., Vanderveken O.M., Tsuda H., Marklund M., Gagnadoux F., Kushida C.A., Cistulli P.A. (2014). Oral appliance treatment for obstructive sleep apnea: An update. J. Clin. Sleep Med..

[108] Benoist L., de Ruiter M., de Lange J., de Vries N. (2017). A randomized, controlled trial of positional therapy versus oral appliance therapy for position-dependent sleep apnea. Sleep Med..

[109] Marklund M. (2017). Update on oral appliance therapy for OSA. Curr. Sleep Med. Rep..

[110] Sheats R.D., Schell T.G., Blanton A.O., Braga P.M., Demko B.G., Dort L.C., Farquhar D., Katz S.G., Masse J.-F., Rogers R.R. (2017). Management of side effects of oral appliance therapy for sleep-disordered breathing. J. Dent. Sleep Med..

[111] Marchetti E., Petro E., Gaggioli F., Lardani L., Mancini L., Marzo G. (2020). The dentist’s role in diagnosis and treatment of obstructive sleep apnea syndrome: A literature review. J. Biol. Regul. Homeost. Agents.

[112] Levendowski D.J., Morgan T., Westbrook P. (2012). Initial evaluation of a titration appliance for temporary treatment of obstructive sleep apnea. J. Sleep Disord. Ther..

[113] Padma A., Ramakrishnan N., Narayanan V. (2007). Management of obstructive sleep apnea: A dental perspective. Indian J. Dent. Res..

[114] Conley R.S. (2011). Evidence for dental and dental specialty treatment of obstructive sleep apnoea. Part 1: The adult OSA patient and Part 2: The paediatric and adolescent patient. J. Oral Rehabil..

[115] Zucconi M., Caprioglio A., Calori G., Ferini-Strambi L., Oldani A., Castronovo C., Smirne S. (1999). Craniofacial modifications in children with habitual snoring and obstructive sleep apnoea: A case-control study. Eur. Respir. J..

[116] Guarda-Nardini L., Manfredini D., Mion M., Heir G., Marchese-Ragona R. (2015). Anatomically Based Outcome Predictors of Treatment for Obstructive Sleep Apnea with Intraoral Splint Devices: A Systematic Review of Cephalometric Studies. J. Clin. Sleep Med..

[117] Vale F., Albergaria M., Carrilho E., Francisco I., Guimarães A., Caramelo F., Maló L. (2017). Efficacy of Rapid Maxillary Expansion in the Treatment of Obstructive Sleep Apnea Syndrome: A Systematic Review With Meta-analysis. J. Evid.-Based Dent. Pract..

[118] Camacho M., Chang E.T., Song S.A., Abdullatif J., Zaghi S., Pirelli P., Certal V., Guilleminault C. (2017). Rapid maxillary expansion for pediatric obstructive sleep apnea: A systematic review and meta-analysis. Laryngoscope.

[119] Sheats R.D. (2020). Management of side effects of oral appliance therapy for sleep-disordered breathing: Summary of American Academy of Dental Sleep Medicine recommendations. J. Clin. Sleep Med..

[120] Zaghi S., Holty J.-E.C., Certal V., Abdullatif J., Guilleminault C., Powell N.B., Riley R.W., Camacho M. (2016). Maxillomandibular Advancement for Treatment of Obstructive Sleep Apnea: A Meta-analysis. JAMA Otolaryngol. Head Neck Surg..

[121] Camacho M., Liu S.Y., Certal V., Capasso R., Powell N.B., Riley R.W. (2015). Large maxillomandibular advancements for obstructive sleep apnea: An operative technique evolved over 30 years. J. Cranio-Maxillofac. Surg..

[122] Handler E., Hamans E., Goldberg A.N., Mickelson S. (2014). Tongue suspension: An evidence-based review and comparison to hypopharyngeal surgery for OSA. Laryngoscope.

[123] Senaratna C.V., Perret J.L., Lodge C.J., Lowe A.J., Campbell B.E., Matheson M.C., Hamilton G.S., Dharmage S.C. (2017). Prevalence of obstructive sleep apnea in the general population: A systematic review. Sleep Med. Rev..

[124] Wang X., Ouyang Y., Wang Z., Zhao G., Liu L., Bi Y. (2013). Obstructive sleep apnea and risk of cardiovascular disease and all-cause mortality: A meta-analysis of prospective cohort studies. Int. J. Cardiol..

[125] Javaheri S., Barbe F., Campos-Rodriguez F., Dempsey J.A., Khayat R., Javaheri S., Malhotra A., Martinez-Garcia M.A., Mehra R., Pack A.I. (2017). Sleep Apnea: Types, Mechanisms, and Clinical Cardiovascular Consequences. J. Am. Coll. Cardiol..

[126] Leng Y., McEvoy C.T., Allen I.E., Yaffe K. (2017). Association of Sleep-Disordered Breathing With Cognitive Function and Risk of Cognitive Impairment: A Systematic Review and Meta-analysis. JAMA Neurol..

[127] Rosenzweig I., Glasser M., Polsek D., Leschziner G.D., Williams S.C., Morrell M.J. (2015). Sleep apnoea and the brain: A complex relationship. Lancet Respir. Med..

[128] Ong C.W., O’Driscoll D.M., Truby H., Naughton M.T., Hamilton G.S. (2013). The reciprocal interaction between obesity and obstructive sleep apnoea. Sleep Med. Rev..

[129] Framnes S.N., Arble D.M. (2018). The Bidirectional Relationship Between Obstructive Sleep Apnea and Metabolic Disease. Front. Endocrinol..

[130] Garbarino S., Guglielmi O., Sanna A., Mancardi G.L., Magnavita N. (2016). Risk of Occupational Accidents in Workers with Obstructive Sleep Apnea: Systematic Review and Meta-analysis. Sleep.

[131] Gupta M.A., Simpson F.C. (2015). Obstructive sleep apnea and psychiatric disorders: A systematic review. J. Clin. Sleep Med..

[132] Araghi M.H., Chen Y.-F., Jagielski A., Choudhury S., Banerjee D., Hussain S., Thomas G.N., Taheri S. (2013). Effectiveness of lifestyle interventions on obstructive sleep apnea (OSA): Systematic review and meta-analysis. Sleep.

[133] Sullivan C.E., Issa F.G., Berthon-Jones M., Eves L. (1981). Reversal of obstructive sleep apnoea by continuous positive airway pressure applied through the nares. Lancet.

[134] Weaver T.E., Grunstein R.R. (2008). Adherence to continuous positive airway pressure therapy: The challenge to effective treatment. Proc. Am. Thorac. Soc..

[135] Marklund M., Verbraecken J., Randerath W. (2012). Non-CPAP therapies in obstructive sleep apnoea: Mandibular advancement device therapy. Eur. Respir. J..

[136] Carvalho B., Hsia J., Capasso R. (2012). Surgical therapy of obstructive sleep apnea: A review. Neurotherapeutics.

[137] Franklin K.A., Anttila H., Axelsson S., Gislason T., Maasilta P., Myhre K.I., Rehnqvist N. (2009). Effects and side-effects of surgery for snoring and obstructive sleep apnea—A systematic review. Sleep.

[138] Sundaram S., Lim J., Lasserson T.J. (2005). Surgery for obstructive sleep apnoea in adults. Cochrane Database Syst. Rev..

[139] Doff M.H.J., Hoekema A., Wijkstra P.J., van der Hoeven J.H., Slater J.J.R.H., de Bont L.G.M., Stegenga B. (2013). Oral appliance versus continuous positive airway pressure in obstructive sleep apnea syndrome: A 2-year follow-up. Sleep.

